# The use of an overtube device to assist in advanced therapeutic procedures in patients with a colostomy

**DOI:** 10.1055/a-2194-4529

**Published:** 2023-11-22

**Authors:** Hameed Rehman, Stefano Sansone, Adolfo Parra-Blanco

**Affiliations:** 1 Nottingham University Hospitals Trust, Nottingham, UK; 2 NIHR Nottingham Biomedical Research Centre, Nottingham University Hospitals NHS Trust, Nottingham UK

Endoscopic therapeutic procedures in patients with a colostomy can be challenging owing to the altered anatomy of the bowel and access difficulties. We report two cases of successful therapeutic colonoscopies in patients with a colostomy using DiLumen (Lumendi, Westport, Connecticut, United States) ( Fig. 1 ), which is a new double-balloon device designed to stabilize the endoscopic position and enhance access to the lesion site 1 .

**Fig. 1:**
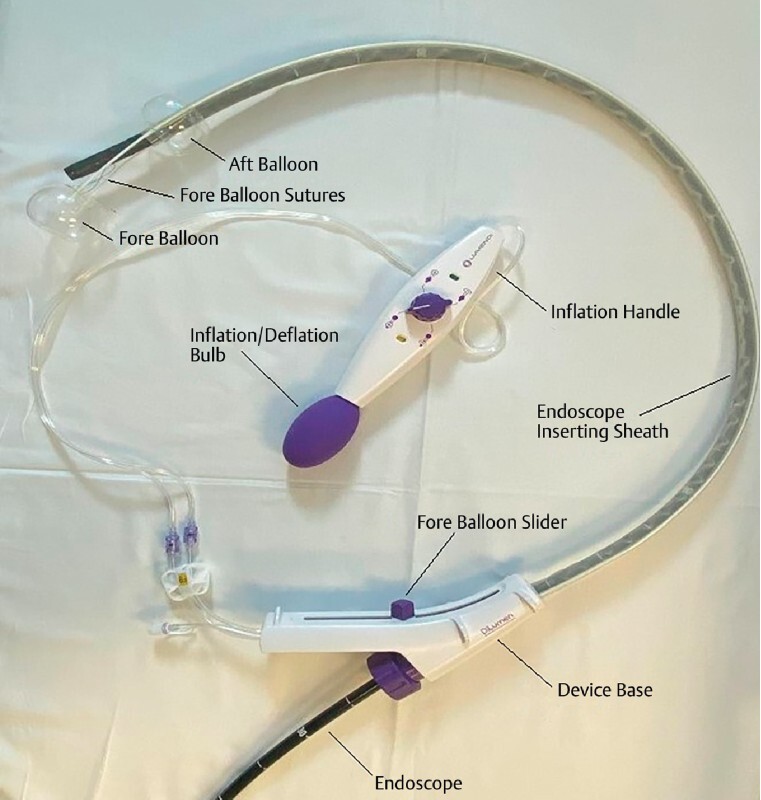
DiLumen device.

**Video 1** Using DiLumen to assist in advanced therapeutic procedures in patients with a colostomy.


The first case involved a 73-year-old man with a colostomy resulting from previous colorectal cancer. He underwent a surveillance colonoscopy that revealed a recurrent 20-mm flat elevated polyp in the ascending colon, located on scar tissue from a previous polypectomy. The polyp was best visualized in a retroflexion position. DiLumen was employed by inflating the balloon between the colostomy and the polyp, ensuring a stable position and preventing air leakage during the polypectomy. The polyp was successfully resected using piecemeal endoscopic mucosal resection (EMR), and an EndoRotor (Interscope, Providence, Rhode Island, USA) was applied to remove small areas of residual polyp. Post-resection, PuraStat (3-D Matrix, Tokyo, Japan) and two clips were utilized to prevent complications. Histology results confirmed high grade dysplasia. The patient is due for a repeat colonoscopy in 3 months.

The second case is of a 53-year-old woman with a colostomy and a previous colonoscopy in which it was not possible to perform polypectomy because of poor bowel distension and air leakage through the colostomy. DiLumen was used to establish stable access and bowel distension by inflating the balloon between the colostomy and the polyp and preventing air leakage. A lifting solution was used in the submucosal area, followed by hybrid EMR with a circumferential incision with the tip of the snare. The polyp was successfully removed with the en bloc technique, and five clips were applied to suture the polypectomy site. Histology confirmed complete resection of the high grade dysplasia polyp.

Endoscopy_UCTN_Code_TTT_1AQ_2AC

## Notes

Endoscopy E-Videos

https://eref.thieme.de/e-videos
*E-Videos* is an open access online section of the journal *Endoscopy* , reporting on interesting cases and new techniques in gastroenterological endoscopy. All papers include a high-quality video and are published with a Creative Commons CC-BY license. Endoscopy E-Videos qualify for HINARI discounts and waivers and eligibility is automatically checked during the submission process. We grant 100% waivers to articles whose corresponding authors are based in Group A countries and 50% waivers to those who are based in Group B countries as classified by Research4Life (see: https://www.research4life.org/access/eligibility/ ). This section has its own submission website at https://mc.manuscriptcentral.com/e-videos
